# Controlling Nutritional Status (CONUT) score as a predictive marker for short-term complications following gastrectomy of gastric cancer: a retrospective study

**DOI:** 10.1186/s12876-021-01682-z

**Published:** 2021-03-05

**Authors:** Feng Sun, Chen Zhang, Zhijian Liu, Shichao Ai, Wenxian Guan, Song Liu

**Affiliations:** Department of Gastrointestinal Surgery, Nanjing Drum Tower Hospital Clinical College of Nanjing Medical University, Nanjing, China

**Keywords:** CONUT, Postoperative complications, Gastric cancer

## Abstract

**Background:**

It is well established that the controlling nutritional status (CONUT) score was correlated with long-term outcomes in gastric cancer (GC), but the significance of CONUT for postoperative short-term outcomes remains unclear. The study aimed to characterize the relationship between CONUT and short-term complications following gastrectomy of GC.

**Methods:**

We collected data on 1479 consecutive GC patients at Nanjing Drum Tower Hospital between January 2016 and December 2018. Univariate and multivariate analyses of predictive factors for postoperative complications were performed. The cutoff value of the CONUT score was determined by Youden index.

**Results:**

Among all of the patients, 431 (29.3%) patients encountered postoperative complications. Multivariate analyses identified CONUT was an independent predictor for postoperative short-term complications (OR 1.156; 95% CI 1.077–1.240; P < 0.001). Subgroup analysis elucidated that CONUT was related to postoperative complications both in early gastric cancer and advanced gastric cancer. We further explored that patients with high CONUT score had prolonged hospital stay (12.3 ± 6.0 vs 11.1 ± 4.6, P < 0.001) and more total hospital charges (7.6 ± 2.4 vs 7.1 ± 1.6, P < 0.001).

**Conclusions:**

The present study demonstrated that the preoperative CONUT was an independent predictor for short-term complications following gastrectomy of GC.

## Background

Gastric cancer (GC) is one of the most common digestive tract cancers worldwide [1]. To this day, radical gastrectomy remains the main option for resectable GC. Despite significant advance has been seen in surgical techniques in recent years, the incidence of complications after radical gastrectomy remains at 15%-25% [2, 3]. Therefore, it is necessary to accurately predict postoperative complications to help perioperative management of GC patients.

Malnutrition is common in GC patients because of a decrease in food intake and energy expenditure. The poor nutritional condition was reported to be correlated with postoperative complications and a worse prognosis [4, 5]. Moreover, immunological status was also regarded as a prognostic marker for GC patients [6, 7]. Several nutritional and inflammatory indicators were monitored routinely before GC surgery, including albumin, total cholesterol, total lymphocytes, hemoglobin, C-reactive protein, neutrophils, and platelet [8–10]. Furthermore, more reliable combined scoring systems were developed to accurately predict patient prognoses, such as neutrophil to lymphocyte ratio (NLR), Prognostic Nutritional Index (PNI), and platelet to lymphocyte ratio (PLR) [8, 11, 12].

Controlling nutritional status (CONUT) score is another nutritional scoring system, covering serum albumin, cholesterol, and lymphocyte counts [13]. Recently, several studies elucidated that CONUT was correlated with the long-term prognosis of different kinds of cancer [14–18]. However, it remains controversial whether CONUT could predict short-term outcomes following tumor resection [19, 20]. Also, little is known about the connection between CONUT and postoperative complications of GC [21]. Therefore, this study intended to characterize the significance of CONUT on short-term complications following gastrectomy of GC.

## Methods

### Patients

We collected data on 1479 consecutive GC patients at Nanjing Drum Tower Hospital between January 2016 and December 2018. All patients underwent curative (R0) gastrectomy and were histologically confirmed. Exclusion criteria include (1) incomplete clinical data; (2) Stage 0 cancer; (3) multi-visceral resection; (4) preoperative radiotherapy or chemotherapy; (5) previous stomach surgery.

### Data collection

The following three types of parameters were extracted: preoperative, intraoperative, and postoperative characteristics. Preoperative index involved age, sex, body mass index, the American Society of Anesthesiologists (ASA) grade, comorbidities (diabetes mellitus, hypertension, and previous abdominal surgery), and laboratory data (neutrophil count, lymphocyte count, platelet count, serum albumin, C-reactive protein, and total cholesterol). The intraoperative features contained type of resection, surgical approach, operation time, and blood loss. Postoperative characteristics included tumor depth, pTNM stage (7th edition), short-term complications, postoperative stay, and hospital costs. The postoperative short-term complications were defined as morbidity that occurred during hospitalization or within 30 days after surgery. The complications were classified according to the Clavien-Dindo (CD) classification system [22].

### Scoring systems of the general condition

Preoperative immune-nutritional and inflammatory scoring systems, including NLR, PLR, PNI, and CONUT, were examined. The NLR and PLR were brought out by dividing the neutrophil and platelet count by the lymphocyte count, respectively [23]. The PNI was obtained from the following formula: (10 × albumin level [g/dL]) + (0.005 × lymphocyte count [number/mm^3^]) [24]. The CONUT was calculated using serum albumin, total cholesterol concentrations, and total lymphocyte count (Additional file 1: Table S1) [13].

### Statistical analysis

Continuous variables were summarized with means ± SD and compared using Student’s t test or Mann–Whitney U test. Categorical variables were summarized with numbers and compared using the Chi squared test or the Fisher exact test. The correlation between postoperative complications and clinicopathological factors were investigated using univariate and multivariate analysis. Variables that were statistically significant in univariate analysis were selected for further multivariate analysis. Binary logistic regression models (Forward: LR) were performed for multivariate analyses. The cut-off value of the CONUT score was determined by Youden index. All *P* values were two-sided and statistical differences were termed as *P* value < 0.05. All statistical analyses were carried out in SPSS 19.0 (Chicago, IL, USA).

## Results

### Patient characteristics

Details of the patient characteristics were summarized in Table 1. This study enrolled 1479 GC patients, including 1083 (73.2%) male and 396 (26.8%) female. The median age was 60 (range: 21–96 years). Diabetes mellitus and hypertension were present in 130 (8.8%) and 485 (32.8%), respectively. Most patients (n = 1403, 94.9%) underwent open gastrectomy. The number of patients who underwent distal gastrectomy, proximal gastrectomy, total gastrectomy was 613 (41.4%), 162 (11.0%), 704 (47.6%), respectively. The overall incidence of postoperative short-term complications was 29.3% (n = 431).

**Table 1:** Demographic and clinical features of patients

Characteristic	N = 1479	Characteristic	N = 1479
Age (year)	60.4 ± 17.3	ASA ≥ 3	879
Gender (n)		Mode of surgical approach (n)	
Male	1083	Laparoscopic	76
Female	396	Open	1403
BMI (kg/m^2^)	23.0 ± 3.5	Type of resection (n)	
Preoperative comorbidities (n)		Distal gastrectomy	613
Previous abdominal surgery	209	Proximal gastrectomy	162
Diabetes mellitus	130	Total gastrectomy	704
Hypertension	485	Operation time (min)	232.2 ± 61.8
Preoperative laboratory data		Blood loss (ml)	221.7 ± 204.8
Neutrophil count (cell/mm^3^)	3614 ± 1705	Tumor invasion	
Lymphocyte count (cell/mm^3^)	1652 ± 611	T1–2	629
Platelet count (cell/mm^3^)	208,851 ± 69,462	T3–4	850
Serum albumin (g/dL)	3.9 ± 0.3	pTNM stage I/II/III/IV	505/366/592/16
Total cholesterol (mg/dL)	157.1 ± 33.1	Postoperative complications	
CRP (g/L)	6.0 ± 12.5	Positive	431
NLR	2.6 ± 2.6	Negative	1048
PLR	143.0 ± 79.5	Postoperative stay (days)	11.8 ± 5.5
PNI	47.7 ± 5.0	Total hospital charges (10^4^¥)	7.4 ± 2.0
CONUT	2.1 ± 1.6		

BMI = body mass index; CRP = C-reactive protein; NLR = neutrophil-to-lymphocyte ratio; PLR = platelet-to-lymphocyte ratio; PNI = Prognostic Nutritional Index; CONUT = Controlling Nutritional Status; ASA = American Society of Anesthesiologists

### Risk factors correlated with postoperative short-term complications

As shown in Table 2, univariate analyses elucidated that postoperative short-term complications were associated with age, sex, low serum albumin, low total cholesterol, PLR, PNI, CONUT score, type of resection (proximal gastrectomy vs. total gastrectomy), and operation time. Multivariate analyses further revealed that age, gender, CONUT score, type of resection (proximal gastrectomy vs. total gastrectomy), and operation time were independent risk factors for postoperative short-term complications.

**Table 2:** Univariate and multivariate analyses of risk factors associated with postoperative complications

Characteristics	Univariate			Multivariate		
	OR	95% CI	P	OR	95% CI	P
Age	1.014	1.004–1.025	0.008	1.011	1.000–1.023	0.046
Gender						
Male	0.763	0.595–0.977	0.032	0.680	0.525–0.880	0.003
Female						
BMI	0.987	0.956–1.020	0.447			
Preoperative comorbidities						
Previous abdominal surgery	1.003	0.727–1.383	0.988			
Diabetes mellitus	1.133	0.768–1.670	0.529			
Hypertension	1.136	0.896–1.440	0.291			
Preoperative laboratory data						
Neutrophil count	1.000	1.000–1.000	0.843			
Lymphocyte count	1.000	1.000–1.000	0.054			
Platelet count	1.000	1.000–1.000	0.373			
Serum albumin	0.555	0.396–0.776	0.001			
Total cholesterol	0.995	0.992–0.998	0.004			
CRP	1.008	1.000–1.017	0.061			
NLR	1.024	0.983–1.068	0.252			
PLR	1.002	1.000–1.003	0.018			
PNI	0.961	0.939–0.983	0.001			
CONUT	1.158	1.081–1.239	< 0.001	1.156	1.077–1.240	< 0.001
ASA ≥ 3	1.039	0.827–1.307	0.740			
Mode of surgical approach						
Laparoscopic	0.688	0.397–1.195	0.184			
Open						
Type of resection						
Distal gastrectomy	1.204	0.947–1.531	0.129	1.266	0.989–1.621	0.061
Proximal gastrectomy	1.542	1.073–2.215	0.019	1.565	1.081–2.264	0.018
Total gastrectomy						
Operation time	1.002	1.000–1.004	0.016	1.003	1.001–1.005	0.003
Blood loss	1.000	1.000–1.001	0.094			
Tumor invasion (T3–4)	1.196	0.952–1.504	0.124			
pTNM stage (≥ III)	1.039	0.827–1.304	0.743			

BMI = body mass index; CRP = C-reactive protein; NLR = neutrophil-to-lymphocyte ratio; PLR = platelet-to-lymphocyte ratio; PNI = Prognostic Nutritional Index; CONUT = Controlling Nutritional Status; ASA = American Society of Anesthesiologists; OR = odds ratio; CI = confidence interval

We further explored the relationship between postoperative short-term complications and CONUT score in different pathological stages. Based on the Youden index, the appropriate cut-off value was 2 for the CONUT score. Patients were categorized into two groups: high CONUT score (≥ 2), low CONUT score (< 2). As shown in Table 3, Postoperative complications were more frequent in high CONUT group in stage I/II/III. From another perspective, the CONUT score was associated with postoperative complications both in early gastric cancer and advanced gastric cancer.

**Table 3:** Relationship between postoperative complications and CONUT in different stages

	All (n = 1479)	Postoperative complications		P
		Yes (n = 431)	No (n = 1048)	
pTNM stage I				0.007
CONUT < 2	267	59	208	
CONUT ≥ 2	238	78	160	
pTNM stage II				0.011
CONUT < 2	138	32	106	
CONUT ≥ 2	228	82	146	
pTNM stage III				0.006
CONUT < 2	219	51	168	
CONUT ≥ 2	373	127	246	
pTNM stage IV				1.000
CONUT < 2	3	0	3	
CONUT ≥ 2	13	2	11	
EGC				0.005
CONUT < 2	234	51	183	
CONUT ≥ 2	188	64	124	
AGC				< 0.001
CONUT < 2	393	91	302	
CONUT ≥ 2	664	225	439	

CONUT = Controlling Nutritional Status, EGC = early gastric cancer; AGC = advanced gastric cancer

### Preoperative CONUT score as a predictor for postoperative complications

Of the 1479 patients, 431 (29.3%) encountered postoperative short-term complications, and 64 (4.3%) encountered major complications (grade III or more). The details of postoperative complications were presented in Table 4. Postoperative complications were more frequent in high CONUT group than low CONUT group (33.9% vs 22.6%; p < 0.001). Major complications were also more frequent in the high CONUT group (5.4% vs 2.9%; p = 0.018). Congruently, patients with high CONUT scores had prolonged hospital stay (12.3 ± 6.0 vs 11.1 ± 4.6, P < 0.001) and more hospital costs (7.6 ± 2.4 vs 7.1 ± 1.6, P < 0.001).

**Table 4:** Comparisons of postoperative complications associated with CONUT

Characteristics	All (n = 1479)	CONUT		P
		< 2 (n = 627)	≥ 2 (n = 852)	
Overall (n)	431	142	289	< 0.001
Grade I (n)	230	89	141	0.217
Fever > 37.5 °C	141	50	91	
Emesis	153	57	96	
Pain	29	10	19	
Abdominopelvic collection	1	0	1	
Pleural effusion	4	1	3	
Grade II (n)	137	35	102	< 0.001
Blood transfusions	59	8	51	
Early postoperative bowel obstruction	2	0	2	
Gastroparesis	25	10	15	
Liver-function abnormalities	1	1	0	
Wound infection	8	1	7	
Pneumonia	26	7	19	
Intra-abdominal infections	17	5	12	
Urinary tract infection	4	1	3	
Enteritis	3	0	3	
Bacteremia	13	4	9	
Grade III (n)	58	17	41	0.040
Anastomotic leakage	22	6	16	
Lymphatic leakage	8	2	6	
Pancreatic fistula	2	1	1	
Biliary fistula	1	1	0	
Bleeding	8	2	6	
Abdominopelvic collection	1	0	1	
Pleural effusion	8	5	3	
Intra-abdominal abscess	1	1	1	
Wound disruption	3	0	3	
Delayed wound healing	3	0	3	
Gastroparesis	2	0	2	
Early postoperative bowel obstruction	1	0	1	
Splenic necrosis	1	1	0	
Grade IV (n)	3	1	2	1.000
Heart failure	1	0	1	
Brain infarction	1	1	0	
MODS	1	0	1	
Grade V (n)	3	0	3	0.267
Grade ≥ III (n)	64	18	46	0.018
Postoperative stay (days)	11.8 ± 5.5	11.1 ± 4.6	12.3 ± 6.0	< 0.001
Total hospital charges (10^4^¥)	7.4 ± 2.0	7.1 ± 1.6	7.6 ± 2.4	< 0.001

CONUT = Controlling Nutritional Status; MODS = multiple organ dysfunction syndrome

## Discussion

In this study, we evaluated the predictive ability of various nutritional and inflammatory parameters for short-term complications following gastrectomy of GC, including NLR, PLR, PNI, and CONUT. Only CONUT was found to be statistically correlated with postoperative complications both in univariate and multivariate analyses. These findings revealed that a high CONUT score was a strong predictor for postoperative short-term complications.

CONUT was initially proposed to assess patients' nutritional conditions in 2005 by Gonzalez and colleagues [13]. It was obtained from serum albumin concentration, total cholesterol concentration, and total lymphocyte count. The easy-to-use index could provide a prompt and accurate preoperative evaluation. It has been well established that preoperative CONUT served as a prognostic factor for long-term outcomes in several cancers, including renal cell cancer, hepatocellular cancer, lung cancer, and colorectal cancer [25–29]. As for GC, recent studies also elucidated that CONUT was significantly related to long-term prognosis [14, 30–32]. However, the correlation between CONUT and short-term outcomes remained unclear because the number of relevant studies was small [4, 21]. Ryo et al*.* recently reported that stage II/III GC patients with high preoperative CONUT scores were more likely to suffer postoperative pneumonia [31]. Li et al*.* elucidated that high CONUT was related to severe postoperative complications [33]. The present study thoroughly accessed the ability of the preoperative CONUT in predicting short-term outcomes following gastrectomy. Our results suggest that the incidence of postoperative overall complications or major complications was higher in patients with high CONUT scores. Furthermore, prolonged hospital stay and more total hospital charges were also observed in patients with high CONUT scores. Therefore, the current study shed light on that the CONUT score might be a useful predictor, not only for long-term outcomes but also for short-term outcomes in GC patients.

The biological mechanism why CONUT could be a predictor for short-term outcomes in GC has not been clearly understood. Here we tried to explain the reasons from each parameter of CONUT. Firstly, serum albumin, representing protein metabolism, is a strong marker of nutritional status. Low serum albumin is not only correlated with poor prognosis in GC but also reported to be a promising predictor for short-term outcomes following gastrectomy [10, 34, 35]. Gunder et al*.* recently reported that albumin levels were better to predict both short-term and long-term GC patient outcomes than complex parameters (PNI, NLR, PLR, and SII) [36]. Serum albumin level was so significant that it has twice the weight of the other components in the CONUT score. Multivariate analysis revealed that the CONUT rather that albumin level was correlated with postoperative short-term complications. Secondly, total cholesterol level, representing lipid metabolism, has been found to be related to tumor progression and overall survival in various kinds of cancer [37, 38]. Several studies suggested that a low cholesterol level might affect the antioxidant reserve and inflammatory response [39, 40]. Finally, total lymphocyte, representing host immunocompetence, was also reported to be correlated with the prognosis of GC patients [41]. The combination of these three components into CONUT allows different phases of nutrition to be included, which enhances the accuracy to assess general conditions.

We acknowledged some potential limitations in this study. First, it was a retrospective, single-center study. Patients enrolled were from just one institution and were ethnically homogeneous. Second, the potential factors influencing preoperative immune-nutritional status were not accessed, such as cancer-related inflammation, chronic renal failure, and liver cirrhosis. Third, the suitable cutoff value for the CONUT score was not yet unified. Therefore, more studies are warranted for elucidating the predictive ability of CONUT for postoperative complications of GC patients.

## Conclusion

In conclusion, the present study demonstrated that the preoperative CONUT was an independent predictor for short-term complications following gastrectomy of GC.

## Supplementary Information

**Additional file 1**. Definition of CONUT.

## Data Availability

The datasets analyzed during the current study are available from the corresponding author on reasonable request.

## Abbreviations

BMI: Body mass index
CRP: C-reactive protein
NLR: Neutrophil-to-lymphocyte ratio
PLR: Platelet-to-lymphocyte ratio
PNI: Prognostic Nutritional Index
CONUT: Controlling Nutritional Status
ASA: American Society of Anesthesiologists
