# Supplementary material for: Controlling Nutritional Status (CONUT) score as a predictive marker for short-term complications following gastrectomy of gastric cancer: a retrospective study
Source: BMC Gastroenterol. 2021 Mar 5;21:107. doi: 10.1186/s12876-021-01682-z (PMC7934386; doi:10.1186/s12876-021-01682-z)
Supplement: Supplementary file 1 — Additional file 1. Definition of CONUT. [file 12876_2021_1682_MOESM1_ESM.docx]

**Title:** Controlling Nutritional Status (CONUT) score as a predictive marker for short-term complications following gastrectomy of gastric cancer: a retrospective study

**Running title:** SUN *et al*: CONUT as a predictor for postoperative short-term complications in GC

**Authors:** Feng Sun^*^, Chen Zhang^*^, Zhijian Liu, Shichao Ai, Wenxian Guan^🖂^, Song Liu^🖂^

Department of Gastrointestinal Surgery, Nanjing Drum Tower Hospital, the Affiliated Hospital of Nanjing University Medical School

**^*^**The authors contributed equally to this work.

^🖂^**Correspondence:** Wenxian Guan and Song Liu, Department of Gastrointestinal Surgery, Nanjing Drum Tower Hospital, the Affiliated Hospital of Nanjing University Medical School. Email: medguanwx@163.com; medical.lis@gmail.com

**Supplemental Table 1. Definition of CONUT**

|  | CONUT | | | |
| --- | --- | --- | --- | --- |
| Parameters | Normal | Light | Moderate | Severe |
| Serum albumin (g/dL) | 3.5-4.5 | 3.0-3.49 | 2.5-2.9 | <2.5 |
| Score | 1 | 2 | 4 | 6 |
| Total lymphocyte (count/mm^3^) | ≥1600 | 1200-1599 | 800-1199 | <800 |
| Score | 0 | 1 | 2 | 3 |
| Total cholesterol (mg/dL) | >180 | 140-180 | 100-139 | <100 |
| Score | 0 | 1 | 2 | 3 |
| Total score | 0-1 | 2-4 | 5-8 | 9-12 |
